# The role of intrinsically disordered regions of SARS-CoV-2 nucleocapsid and non-structural protein 1 proteins

**DOI:** 10.3389/fchem.2025.1597656

**Published:** 2025-06-11

**Authors:** Getasew Shitaye, Nataliia Ventserova, Gianluca D’Abrosca, Martina Dragone, Eunice Wairimu Maina, Roberto Fattorusso, Rosa Iacovino, Luigi Russo, Carla Isernia, Gaetano Malgieri

**Affiliations:** ^1^ Department of Environmental, Biological and Pharmaceutical Sciences and Technologies, University of Campania “Luigi Vanvitelli”, Caserta, Italy; ^2^ Department of Biomedical Sciences, College of Medicine and Health Sciences, Bahir Dar University, Bahir Dar, Ethiopia; ^3^ Department of Human Sciences, Link Campus University, Roma, Italy; ^4^ Interuniversity Research Centre on Bioactive Peptides, University of Naples “Federico II”, Naples, Italy

**Keywords:** intrinsically disordered proteins, coronaviruses, SARS-COV-2 variants, protein-protein interactions, host-viral interactions

## Abstract

Virus survival inside the host cell depends on the intricate mechanisms that recruit proteins involved in the arms race. Severe Acute Respiratory Syndrome Coronavirus-2 (SARS-CoV-2) proteome exhibits important levels of structural order. However, some of the SARS-CoV-2 proteins, such as the Nucleocapsid (N) and Non-structural protein 1 (Nsp1), contain a considerably significant amount of intrinsically disordered regions (IDRs) that play indispensable roles in the intra-viral and virus-host interaction. Here, focusing on proteins that contain a relevant percentage of IDRs, we discuss experimental and computational studies sought to support IDRs as a key player in the interplay with ordered domains, the biological role as potential origin for variants of SARS-CoV-2, and their association with virus transmissibility. Furthermore, we also highlight the potential involvement of IDRs in the viral-host protein interaction and host cellular machinery. Thus, shading lights on the dark proteome of the virus and looking for therapeutic approaches beyond the classic structure-function paradigm may contribute to the efforts sparking the quest for therapeutics.

## 1 Introduction

One of the central dogmas of molecular biology is the sequence-structure-function relationship: the primary sequence of amino acids determines the protein’s three-dimensional structure that, in turn, defines its function. In the past couple of decades, such a relationship in the field of structural biology has been questioned by the fact that naturally occurring unstructured proteins that deviate from this dogma are increasingly identified. The so-called Intrinsically Disordered Proteins (IDPs), which do not have well-defined secondary, tertiary and, in specific cases, quaternary structures but still exhibit many important biological processes (Dyson and Wright, 2005; Dunker et al., 2001; Kulkarni et al., 2025), have become hot topics in the “proteins world.” Just like other functional molecules, disordered proteins play key roles in molecular and cellular events such as cellular signal transduction, cell cycle, transcriptional regulation, and translation (Tompa, 2002; Iakoucheva et al., 2002; Mishra et al., 2020). Moreover, a great number of proteins that are not commonly classified as IDPs encompass extensive segments characterized by an intrinsic disorder, often defined as intrinsically disordered regions (IDRs). These IDRs are widely distributed across the proteome of different organisms with significant variations within the entire protein in terms of composition and percentage content (Dunker et al., 2001; Peng et al., 2015).

Despite the fact that disordered regions act as mediators of cell signaling and other indispensable biological processes, generally, their essential role has a biological cost: IDRs, not protected by a structure, are more prone to involvement in mis-functional processes, i.e., aggregation above all.

Physico-chemical changes in the cellular environment can, in principle, affect their normal functions consequently leading to pathogenesis and diseases.

One of the hallmarks of many viruses is the effective utilization of their own IDPs/IDRs to perform essential cellular processes to escape from the host defense mechanism (Davey et al., 2011; Dyson and Wright, 2018). This outlines the importance of a thorough characterization of the role of IDRs to understand the molecular mechanism of diseases beyond the classic structure−function paradigm.

Even though the characterization of IDPs and IDRs is a major challenge in biology, since the outbreak of Coronavirus disease 2019 (COVID-19) tremendous efforts have been made to unravel the structural biology of IDRs in the SARS-CoV-2 proteome. A plethora of computational and experimental spectroscopic techniques such as Nuclear Magnetic Resonance (NMR), Circular Dichroism (CD) and Small-angle scattering of X-rays (SAXS), have been used to study these dancing proteins. Beyond reviewing the common knowledge on IDPs and IDRs, the aim of this review is summarizing and analyzing the intrinsically disordered regions of the SARS-CoV-2 proteome, the information available on their biological role, their interaction with globular domains, their importance as source of mutations and viral transmissibility, and the potential involvement with the host cellular machineries. Firstly, we highlighted the percentage of the intrinsic disordered regions within different proteins across the SARS-CoV-2 proteome and opted to summarize the role of IDRs in Nucleocapsid and Non-structural protein 1. We then discuss the role or association of IDRs with SARS-CoV-2 infectivity and transmissibility along with experimentally and computationally explored molecular driving forces acting as local determinants of IDRs conformational changes. Finally, focusing on the SARS-CoV-2 proteins that contain significant disordered regions, we highlighted the potential involvement of their IDRs in the viral-host protein interactions and the effects on the host cellular machinery.

## 2 Overview of genome organization and IDR content of coronaviruses

Coronaviruses (CoVs) are a widely varied family of enveloped positive-sense single-stranded RNA viruses. By virtue of their ubiquitous presence in nature, they are broadly spread among mammals and avian species. CoVs belong to the *Coronaviridaes* family of the *Nidovirales* order and can be classified into four genera: alphacoronavirus, betacoronavirus, gammacoronavirus and deltacoronavirus.

Betacoronavirus (β-CoV), such as Severe Acute Respiratory Syndrome Coronaviruses (SARS-CoV-1 and SARS-CoV-2) and Middle Eastern Respiratory Syndrome virus (MERS-CoV), are highly pathogenic viruses for humans that share similar genome and replication strategies (Enjuanes et al., 2006; V’kovski et al., 2021).

The SARS-CoV-2 genome, with an approximate length of 30 kb, encodes structural proteins, non-structural proteins (NSPs), and a number of accessory proteins. The four major structural proteins, namely, Spike (S), Nucleocapsid (N), Membrane (M), and Envelope (E) protein, are essential components required for the production of a structurally complete viral particle. Specific interactions between the viral S protein and host cell surface factors, serine protease TMPRSS2, and the receptor angiotensin-converting enzyme 2 (ACE2) promote viral uptake and cellular fusion. N protein is implicated in RNA encapsidation, while proteins E and M guarantee RNA genome (+ssRNA) incorporation into viral particles during the assembly process.

**FIGURE 1:**
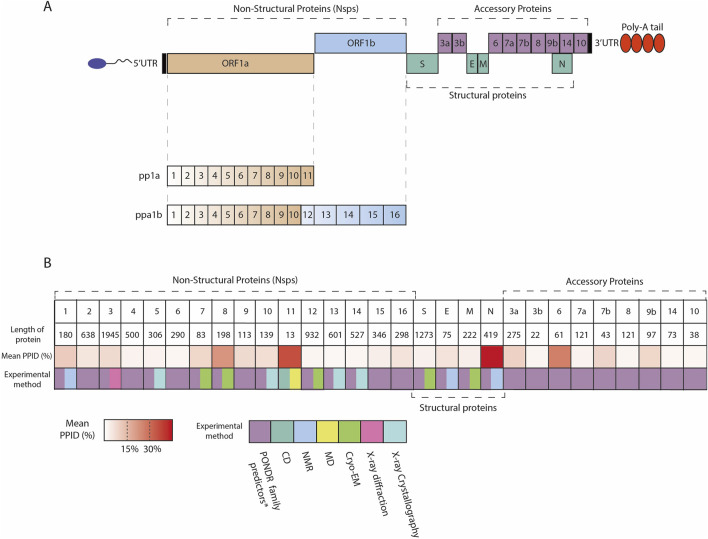
**(A)** Scheme of SARS-CoV-2 genome organization; SARS-CoV-2 contains 5′capped mRNA that has a leader sequence (LS), poly-A tail at 3′end, and 5′and 3′UTR. It contains genes such as ORF1a, ORF1b, Polyproteins (pp1a and pp1ab), S: Spike, ORF3a, ORF3b, E: Envelope, M: Membrane, ORF6, ORF7a, ORF7b, ORF8, ORF9b, ORF14, N: Nucleocapsid, and ORF10. **(B)** Overview of the IDRs across SARS-CoV-2 proteome. Heatmap indicates the mean PPID predicted by Giri et al. (2021) and experimental methods used to characterize SARS-CoV-2 proteins structure. The mean PPID of the all protein except Nsp11 is taken from Giri et al. (2021), whereas the level of disorder for Nsp11 is described by Gadhave et al. (2021). *Giri et al. used the translated sequences of SARS-CoV-2 proteins [GenBank database Clark et al. (2016) (Accession ID: NC_045512.2)], and predicted IDPRs using a range of bioinformatics tools such as PONDR® (Predictor of Natural Disordered Regions) family including PONDR ®VLS2 Peng et al. (2006), PONDR ®VL3 Peng et al. (2005), PONDR ®FIT Xue et al. (2010), and PONDR ®VLXT Romero et al. (2001), as well as the IUPred platform for predicting long (≥30 residues) and short IDPRs (<30 residues) Mészáros et al. (2018).

Upon infection, the incoming viral RNA is uncoated inside the host cytosol and two large viral open reading frames, ORF1a and ORF1b, are translated into two polyproteins, pp1a and pp1ab, that are further cleaved into 11 and 15 Nsps, respectively. The 16 Nsps thus produced form the viral replication and transcription complex (Figure 1) (V’kovski et al., 2021; Masters, 2006; Wang et al., 2017; McBride et al., 2014; Malone et al., 2022).

Bioinformatics studies analyzed the percentage of intrinsic disorder (PID), quantified as the ratio between residues predicted to be disordered and the total number of residues, in the MERS-CoV proteome and in individual proteins derived from the MERS-CoV genome. Predicted percentage of intrinsic disorder (PPID) is a metric calculated as the ratio of disordered residues (those predicted to be disordered) to the total number of residues, expressed as a percentage, which is used to estimate the degree of intrinsic disorder within a protein (Rajagopalan et al., 2011; Redwan et al., 2019). Thus, the levels of PPID were applied to categorize proteins as highly ordered (PPID <10%), moderately disordered (10% ≤ PPID <30%), and highly disordered (PPID ≥30%) (Rajagopalan et al., 2011; Giri et al., 2021). According to these examinations, N proteins are characterized by a higher value of PID (Goh et al., 2020; Goh et al., 2013; Alshehri et al., 2021).

In the scenario of SARS-CoV-2, a significant (refers to the percentage) level of disorder content was found for the S1 subunit of S protein and up to 51% for N protein. In these intrinsically disordered regions an abundance of mutations occurring in the 14 major variants were localized [*Variants of Concern (VOC)]: Alpha, Beta, Gamma, Delta, Omicron BA.1 and BA.2; Variants of Interest (VOI) Lambda, Mu, Epsilon, Zeta, Eta, Theta, Iota and Kappa.)* that could favor the virus with benefits for genetic and antigenic drift (Anjum et al., 2022; Quaglia et al., 2022). Another study buttresses that N protein possesses a predicted PID value of more than 60% and demonstrates moderate content of disorder for some of the nonstructural proteins and accessory proteins, namely, Nsp8, Nsp1, ORF6, ORF9b (Giri et al., 2021; Anjum et al., 2022; Rezaei et al., 2022). Interestingly, the nonstructural proteins, including Nsp2-Nsp6, Nsp9, Nsp10, and Nsp12-Nsp16 of SARS-CoV-2 proteins, show a quite less content of disordered regions (Giri et al., 2021; Anjum et al., 2022).

### 2.1 The nucleocapsid protein

**FIGURE 2:**
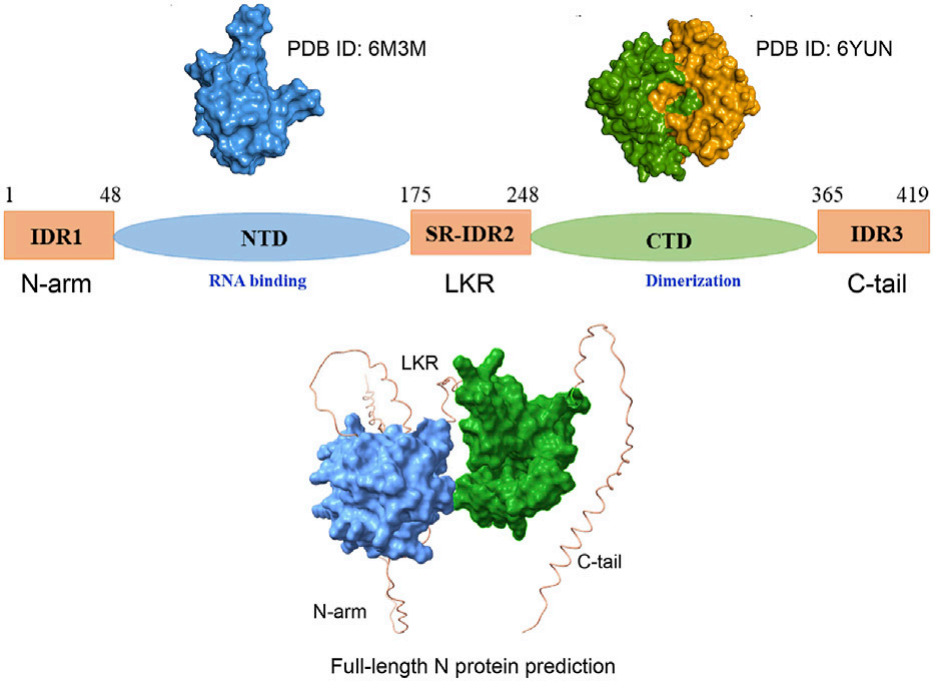
Scheme of the overall structure of the SARS-CoV-2 N protein. N protein consists of the N-terminal arm (IDR1), the RNA-binding N-terminal domain (NTD), the central linker region (LKR) within a Ser/Arg (SR)–rich motif, the C-terminal domain (CTD), associated with dimerization, and the C-terminal tail (C-tail). The up-left panel illustrates the surface of the amino-terminal (N-terminal) domain (PDB ID: 6M3M) Kang et al. (2020). The up-right panel illustrates the surface representation of the dimeric carboxy-terminal (C-terminal) domain (PDB ID: 6YUN)) (each monomer presented by separate color code). Zinzula et al. (2021). The lower panel represents AlphaFold 3 prediction of full-length N protein encompassing folded domain (surface representation) and IDRs Abramson et al. (2024).

As previously discussed, within the SARS-CoV-2 proteome, the N protein stands out with a substantial amount of predicted disordered content. The SARS-CoV-2 N is a 419 amino acid protein that bears two ordered domains: the RNA-binding N terminal domain (NTD) (residues 48–175), which contains the RNA recognition motif (RRM), and the C terminal domain (CTD) (248–365) (Kang et al., 2020; Jayaram et al., 2006; Grossoehme et al., 2009; Huang et al., 2004), primarily associated with the formation of higher-ordered structures. The N protein also contains three intrinsically disordered regions: IDR1 (residues 1–48), the central SR-IDR2 (residues 175–248), which is enriched with serines and arginines and separates the two ordered domains, and the C-terminal IDR. Interestingly, the N in complex with its partner proteins Nsp3a exhibits dynamic nature (Figure 2). For example, as clearly demonstrated from its solution NMR structure (PDB ID: 7PKU), the SR-IDR2 shows a tendency to form a helix (^219^LALLLLDRLNQL^230^) and disordered polar strand (^243^GQTVTKKSAAEAS^255^) (Bessa et al., 2022). N shows structural plasticity and its predicted structure could sample modest helical conformation based on the sequence ^390^ QTVTLLPAADLDDFSKQLQQSMSSADSTQA^419^ (IDR3 region) (Zhao et al., 2022).

A great number of studies have shown that several N proteins bind to multiple RNA molecules without exhibiting binding specificity to RNA sequences (Carlson et al., 2020; Iserm et al., 2020; Perdikari et al., 2020; Savastano et al., 2020). It is assumed that a single molecule of genomic RNA is enveloped by multiple N proteins and packaged into virions (Yao et al., 2020; Cubuk et al., 2021; Klein et al., 2020).

**FIGURE 3:**
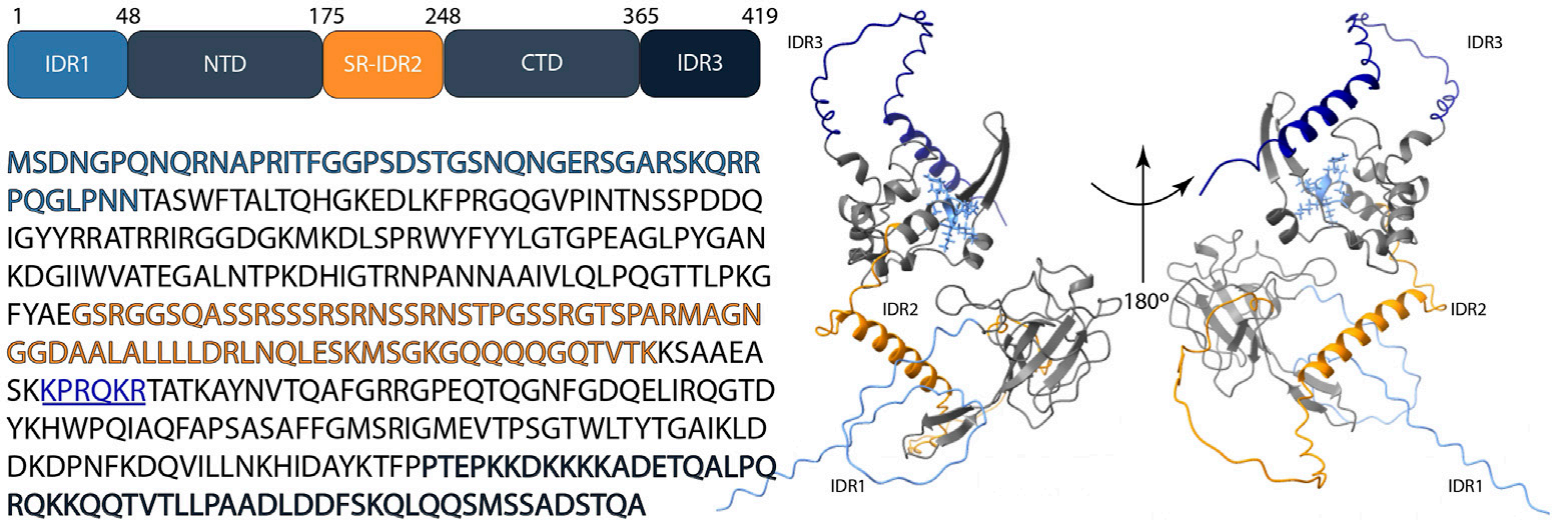
Schematic representation of the structure of the SARS-CoV-2 N protein; N-terminal domain (NTD), C-terminal domain (CTD), IDR1 (light blue color), SR-IDR2 (orange color), IDR3 (dark blue color) and the stretch of positively charged residues: Lys257- Arg262 (underlined, illustrated in blue color with side-chain visualization on the protein structure). The 3D structure shows the prediction by AlphaFold 3 based on the sequence (accession no: P0DTC9.1) Abramson et al. (2024).

*In vivo* studies showed that the recognition of RNA that has to be packed requires both the NTD and CTD domains of N protein (Woo et al., 2019); specifically, the N-terminal linker region and C-terminal segments are required for RNA recognition and binding. (Jayaram et al., 2006; Grossoehme et al., 2009; Cui et al., 2015; Yu et al., 2005; Nelson and Stohlman, 1993) (Cubuk et al., 2021; Chang et al., 2009; Wu et al., 2021). By means of charge distribution analysis of dimeric CTDs, Jia et al. identified in their surface a stretch of positively charged residues (between Lys257 and Arg262) involved in RNA-binding (Jia et al., 2022) and conserved among SARS-CoV-1, SARS-CoV-2, and MERS-CoV (Figure 3).

Mutations of these conserved positive residues can significantly decrease the binding affinity with RNA, confirming their role in RNA binding (Chen et al., 2007; Hsin et al., 2018; Yang et al., 2021). Upon binding to the RNA, the SARS-CoV-2 N protein undergoes a more compact conformation compared to its free form (Ribeiro-Filho et al., 2022). Interestingly, the stability of the small N protein structure seems to be dependent on the N-terminal RNA-binding domain (Sekine et al., 2023). Inhibiting the RNA binding specificity of N proteins in betacoronaviruses with antiviral drugs could combat a range of these viruses, aiding in future outbreaks.

### 2.2 Non-structural protein 1 (Nsp1)

**FIGURE 4:**
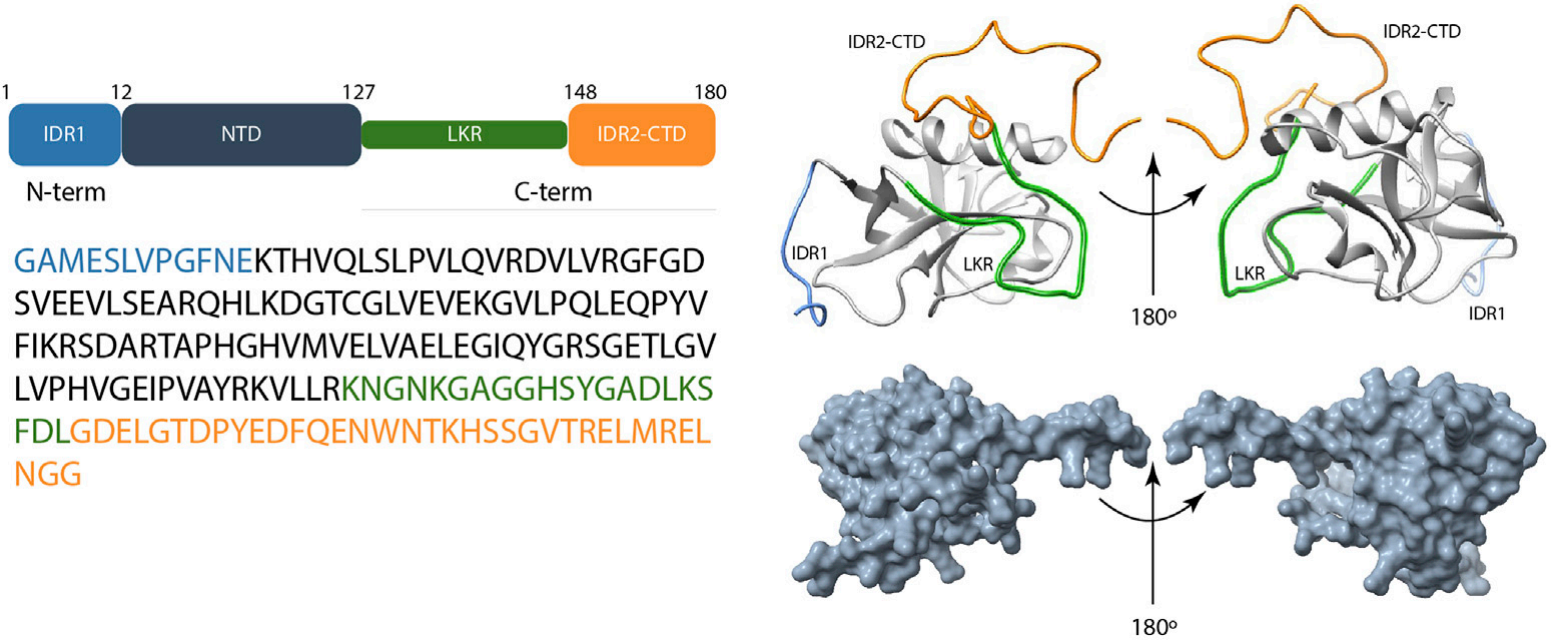
Scheme of the overall structure of the SARS-CoV-2 Nsp1 and its disordered predicted regions. Left panel shows the schematic representation of domain organization and amino acid sequence of Nsp1. Up-right panel shows experimental structure of Nsp1 that consists of IDR1 (light blue color), the N-terminal domain (NTD), LKR (Linker) (green color), and the IDR2-CTD (orange color) (the last two usually reported under the denomination C-terminal tail) (PDB ID:8AOU). Wang et al. (2023) Right-down panel represents the AlphaFold 3 model of Nsp1 structure Abramson et al. (2024).

Several research delves into the characterization of the full-length SARS-CoV-2 Nsp1 (1–180) (Figure 4), which plays a dual role through initiating viral protein synthesis and inhibiting host translation; it engages with the host 40S ribosomal subunit, thereby prevents initiation of host protein translation. Crystal structures of Nsp1 proteins of SARS-CoV (aa 10–126) (Almeida et al., 2007) and SARS-CoV-2 (aa 12–127) (Clark et al., 2021; Semper et al., 2021) show nearly the same folded N-terminal domain architecture. Interestingly, most of the residues predicted to be disordered are localized in the C-terminal tail (125–179) that directly binds within the mRNA entry tunnel of the 40S (Agback et al., 2021; Ilyas et al., 2025).

2D ^15^N-{^1^H} NOE NMR experiment on full-length SARS-CoV Nsp1 protein revealed that its residues 1 to 12 and 129 to 179 have small positive or negative ^15^N,^1^H heteronuclear NOE values, characterizing these regions as short N-terminal and long C-terminal disordered tails (Almeida et al., 2007) with a remarkable conformational flexibility at sub-nanoseconds timescale. Using cryogenic electron microscopy, different groups of researchers were able to solve the structure of the C-terminal portion of SARS-CoV-2 Nsp1 in complex with the 40S ribosome (Thoms et al., 2020; Schubert et al., 2020). These structures confirm the presence of a short α-helix (P153 to N160) and a second C-terminal α-helix spanning residues S166 to N178. Likewise, the study by Semper et al. combines SARS-CoV-2 Nsp1 (13–127) crystal structure (PDB ID:7K3N) with amino acid sequence analysis, data from prediction tools, and homology-based models, showing the presence of 12 N-terminal residues comprising disordered regions as well as a largely disordered linker between the end of the globular domain and the C-terminal helix-turn-helix (Semper et al., 2021).

Furthermore, a C-terminal helix-turn-helix motif is proposed in their homology-based model of full-length Nsp1 (Semper et al., 2021). While the study of Kumar and coworkers (Kumar et al., 2021), in line with previous studies, validated the C-terminal IDR region (131–180) in isolation, Thoms et al. have shown that after interaction with the 40S ribosome, such region adopts a helical structure (Thoms et al., 2020) suggesting its important role in regulating host mRNA translation.

Secondary structure analysis of the full-length SARS-CoV-2 Nsp1 protein by means of NMR (Agback et al., 2021; Wang Y. et al., 2021) revealed one folded domain that comprises residues T14–L125 and two disordered chains at the N-terminus (residues 1–13) and at the C-terminus (residues 126–180), respectively. As clearly demonstrated by Agback and coworkers (Agback et al., 2021), SARS-CoV-2 Nsp1 in solution shows different dynamics of N- and C-termini. They were also able to demonstrate that residues located between the folded domain and the disordered C-terminal exhibit different conformations. For example, the region at the beginning of the C-terminal domain (^122^LLRK^125^) has been shown to adopt two different conformations distinguishable on the NMR time scale even at high temperature (308K) (Agback et al., 2021).

## 3 Globular domain–IDRs interplay

Along with interplay between the structured part and the IDRs within each specific protein, the vast majority of disordered regions in the SARS-CoV-2 are also involved in protein-protein interactions with other viral and host proteins. Protein-protein interaction (PPI) is a key biological process, established often in a specific and stable manner by a physical contact of two or more proteins in cells which form a complicated network termed “interactome” (Koh et al., 2012; Venkatesan et al., 2009).

**FIGURE 5:**
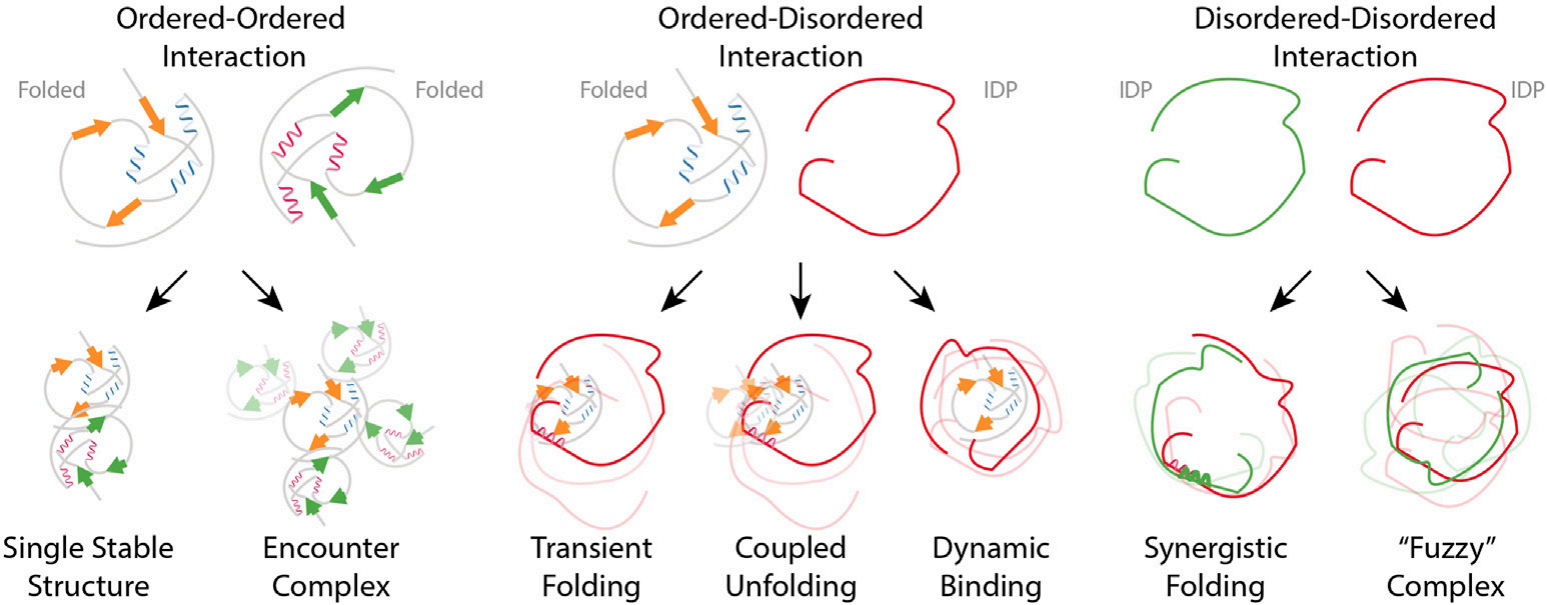
Schematic overview in PPIs (Left panel) Ordered-ordered interaction describes a scenario where two systems possessing well-defined, structured arrangements, engage with each other while maintaining their respective order. (Middle panel) Ordered-disordered interactions refer to the binding of an IDP to a folded protein; the IDP can either stay unfolded in the bound states and adopt multiple conformations or fold upon binding. In the case of coupled unfolding, though the folded binding partner remains structured, it can partially unfold. (Right panel) Interactions between two IDPs through regions that are inherently flexible or disordered can result in “Fuzzy complexes” which exhibit heterogeneous structure without a fixed binding interface Adapted from Spreitzer et al. (2020).

Due to the dynamic nature of proteins, the environment in which they exist largely influences their motion, and it must be taken into proper consideration while discussing protein-protein interactions (PPI) (Figure 5).

IDPs are prone to posttranslational modifications (Darling and Uversky, 2018; Jin and Gräter, 2021; Niklas et al., 2015) and, therefore, are likely to exist as chemically heterogeneous intracellular populations. Compared to the structured proteins, IDPs are more susceptible to environmental changes that can determine the IDR/IDP structure and function. The term “environment” intends a broad context: physicochemical environment (pH, viscosity, pressure, salt concentration, *etc.*) (Lindsay et al., 2021; Verma et al., 2020), biomolecular environment, (interactors and location), physiological environment (cell-type, cell-cycle, stress, *etc.*) (Verma et al., 2020), and intramolecular interactions (Bugge et al., 2021; Bugge et al., 2020).

As well documented from physicochemical (Lin et al., 2018) and quantitative studies (Bowman et al., 2020), amino acid sequence encodes IDRs conformational behavior as well as their interaction and function (Das et al., 2015; Chong and Mir, 2021). Collectively, many studies evidence that polypeptides and proteins that carry high net charges are known to undergo extended conformations. Charged residues in intrinsically disordered proteins also drive the interaction with biomolecules (Bowman et al., 2020; Milorey et al., 2021).

Transient interactions are another attribute that characterize disordered proteins. Multiple interactions contribute to their dynamic behavior and to possible structural transitions. In many cases, hydrophobic interactions primarily play a role in the disorder-structure paradigm of the IDR systems. Abyzov et al. showed liquid−liquid phase separation (LLPS) derived as a result of intermolecular interactions. The interaction was observed to be responsible for modulating internal motions, slowing down the time scales of IDP motions, and restricting the amplitude of the local backbone conformational sampling (Abyzov et al., 2022). In their analysis, the authors noted that the locally accessible surface determines the interaction between IDP tails and the folded domains that in turn act as local determinants of IDR conformational behavior. In their study, Taneja and Holehouse exploited this behavior to investigate the role of the relative position and accessibility of charged residues across the surface of a folded domain. Simulations on homologs protein with variable charge distribution demonstrated that even slight changes in surface charge impact IDR behavior and interaction with folded domains (Taneja and Holehouse, 2021). Of note, due to the presence of multiple charge patches on naturally occurring proteins and their physical accessibility to local IDRs, these changes in the chemical environment are highly relevant to modulate the interaction (Wohl et al., 2025).

Mukherjee et al. used conformation-sensitive single-molecule Förster resonance energy transfer and CD to understand the effects of cellular components on the conformation of the SARS-CoV-2 RG-1 RNA G4Q. The authors confirmed that salts, co-solutes, crowders, and other IDPs affect the conformation of the SARS-CoV-2 RG-1 RNA G4Q. For instance, in the absence of monovalent K^+^ salt, the RG-1 SARS-CoV-2 RNA-G4Q exists as an equilibrium between folded and metastable conformation, whereas at a high concentration of K^+^, only the fully folded conformations are preserved. Recent reports of Parkinson’s disease cases involving young patients after SARS-CoV-2 infections showed that amyloidogenic disordered α-Syn-derived peptides stabilized the folded state of RG-1 G4Q, although human islet amyloid polypeptide (hIAPP) stabilized the partially folded state, leading to the conclusion that these interactions may afflict expression profiles of disease-modifying genes (Mukherjee et al., 2022). Yesudhas et al. used molecular dynamics simulations to investigate the binding contributions of intrinsically disordered residues of S protein to human ACE2 proteins. They observed a disorder-to-order transition (DOT) profile upon binding that is strengthened mainly by the hydrophilic and aromatic residues localized in the region that undergoes such disorder-to-order transition. The authors hypothesized that this more energetically favorable binding of receptor binding domains with ACE2 is due to the positively correlated motion of the DOTs with its neighboring residues (Yesudhas et al., 2021; Akbayrak et al., 2022). Moreover, the conformational disorder of the N- and C-terminal residues and water accessibility of the SARS-CoV-2 envelop protein increased in acidic pH and high calcium ions concentration (Medeiros-Silva et al., 2022).

### 3.1 Nucleocapsid

While considerable emphasis has been placed on the S protein, numerous other proteins of SARS-CoV-2, such as N protein, also hold pivotal significance in viral physiology. However, our understanding of their structural and biophysical characteristics remains comparatively limited. N is an abundant RNA-binding protein involved in packaging and transcription facilitation. It is also one of the mutational hotspots in the SARS-CoV-2 genome.

As reported in Section 2.1, the SARS-CoV-2 N comprises three predicted intrinsically disordered regions, an N-arm (IDR1), a C-tail (IDR3), and a centrally located linker region (SR-IDR2) that connects the NTD and CTD. In their elegant work, Cubuk and co-authors using single-molecule fluorescence spectroscopy with all-atom simulations proved the structural heterogeneity of these three IDR domains (Cubuk et al., 2021).

In betacoronaviruses, the IDR site (the N protein primary phosphorylation site) that acts as a linker between the NTD and CTDdomains has arginine/serine rich region and can undergo phosphorylation, that in turn affects the protein function (Carlson et al., 2020; Peng et al., 2008; Lu et al., 2021). By means of nanosecond fluorescence correlation spectroscopy (FCS), a slower reconfiguration time (τ_r_ = 170 ± 30 ns.) in comparison with other proteins with similar length and charge content was revealed, suggesting that the NTD transiently interacts with the RNA-binding domain. This linker limits the interaction between the NTD and CTD domains but enables them to have an independent function (Cubuk et al., 2021), which probably prevents the formation of NTD dimers. The unstructured central linker can critically affect the N proteins binding conformation to RNA and to other proteins. The concurrent binding of IDR linker to both the CTD and NTD through intra and inter molecular interactions, (Różycki and Boura, 2022), the direct interaction of this linker to RNA *via* electrostatic interaction, and even the phosphorylation process (Carlson et al., 2020; Peng et al., 2008), can cause conformational changes in N protein.

Biomolecular condensates, essential for various cellular processes, can arise from the LLPS containing IDRs and RNA-binding domains. Then, a few studies have shown that, at the body temperature, the S/R-rich central disordered region significantly associates with the RNA-dependent phase separation of N protein where its phosphorylation regulates the condensate viscosity of cells (Iserm et al., 2020; Lu et al., 2021). Additionally, the recent study on the binding and compaction of SARS-CoV-2 full-length N protein and its truncated variants containing the globular domains with and without the linker region (Morse et al., 2023) confirms the pivotal role of the intrinsically disordered regions in maintaining the biological function of N protein. In detail, Morse et al. demonstrate that the N protein region IDR1-NTD-IDR2 missing the CTD and the disordered C-tail shows similar performance to compact the ssDNA as the full-length N protein, whilst the same portion without the IDR2 linker shows a dramatic lower activity. These binding differences to the ssDNA are due to the richness in serines and arginines of the IDR2 region that enables the preservation of the fast on/off kinetics and non-cooperative binding. It is also postulated that IDR2 may provide structural plasticity to the N protein (Zhao et al., 2022).

Experimental studies have quantified the affinity of N protein for RNA. For instance, Wu and his colleagues investigated contribution from each N protein domain to ssRNA binding (Wu et al., 2021). They found that N wild type binds the 20-nt ssRNA (sequence: UUU​CAC​CUC​CCU​UUC​AGU​UU) with high affinity (*K*_*D*_ = 0.007 ± 0.001 μM). In contrast, the isolated N_NTD_ and N_CTD_ have low-affinity binding (*K*_*D*_ = 20 ± 10 and 13 ± 5 μM, respectively). However, inclusion of the LKR region increased RNA binding affinity significantly (0.50 ± 0.08 and 0.35 ± 0.04 μM for N_NTD-LKR_ and N_LKR-CTD_, respectively). Addition of CTD onto NTD-LKR in cis increases the binding affinity to the single-digit nM range (0.006 ± 0.002 μM), but not in trans. Overall this data quantitatively indicates that the wild-type N protein has high-affinity ssRNA binding, and unstructured regions contribute into ssRNA interactions.

The capacity of N protein to undergo LLPS is well-documented, defining starting N protein concentrations equals to 10 µM. However, the droplet sizes did not grow with increased concentration, even at a protein concentration of 40 μΜ. Interestingly, size of condensates was modulated by increasing RNAs concentration (at 1:0.25 protein: RNA ratio, the size and the sphericity of the droplets further increased. Since RNA is involved in the assembly of the viral to form a helical ribonucleoprotein (nucleocapsid), these condensates are proposed to enhance the efficiency of viral replication. Thus, these condensates could become a potential therapeutic target in order to disrupt cycles of SARS-CoV-2 replication (Wang J. et al., 2021).

Moreover, Morse et al. (Morse et al., 2023) suggest a description of a multistep process through which N proteins can bind and package viral RNA (vRNA). In particular, the N protein, which is abundantly expressed in the host cell, forms CTD-mediated dimers and quickly binds vRNA through NTDs and linkers, due to electrostatic interaction. This binding is non-cooperative and fully reversible. Over time, the binding increases resulting in substrate compaction as neighboring proteins multimerize. This assembly mechanism is modulated by the C-terminal domain, which can fully compact the substrate. Note that the ssDNA-protein complex compaction observed in this study is less extensive with respect to vRNA-N protein compaction inside the viral particle.

As reported above, the N-terminal and C-terminal IDRs contribute to the RNA-binding activity of the SARS-CoV-2 N protein. Using a biosafety level-2 cell culture system (Ju et al., 2021), Luo et al. found that deletion of IDR1 completely abolished SARS-CoV-2 production (Luo et al., 2021). Similarly, truncations of IDR1 weaken the RNA binding, as demonstrated by a chromatography study of the nucleoprotein both in RNA-bound states and RNA-free state (Wu et al., 2021), further suggesting the relevant biological role of IDRs cooperation with the folded domains.

Though both the NTD and CTD can bind RNA, practically no direct cooperation is observed between the two domains in RNA binding (Kubinski et al., 2025). Experimental data from combined coarse-grained molecular simulations with data from small-angle X-ray scattering (SAXS) on full-length SARS-CoV-2 N protein evidenced this scenario (Różycki and Boura, 2022). From the simulations, several intra- and inter-chain contacts between the formed hydrophobic α-helix within the IDR2 and the two ordered domains have been observed. This α-helix of IDR2 showed the most frequent degree of contact with the NTD of the same chain than the NTD of the other chain in the N dimer (Różycki and Boura, 2022).

IDR1 in SARS-CoV-2 N protein has been also found to be an indispensable mediator for the interaction between N protein and Ras-GTPase-activating protein SH3-domain binding (G3BP1), where the interaction leads to stress granule (SG) disassembly (Luo et al., 2021). As confirmed by the mutant, the N protein lacking IDR1 impaired its capability to colocalize with G3BP1 whereas deletion of the C-terminus containing homodimerization domain and IDR3 can facilitate the N protein diffusion within droplets. IDR1 initiates the LLPS of the N protein, whereas the C-terminal region stabilizes the condensate, likely by facilitating protein homodimerization. Meanwhile, SARS-CoV-2 N supposedly interacts with G3BP stress granule assembly factor 2, thereby consequently suppressing the integrated stress response and inhibiting type I interferon responses (Kruse et al., 2021; Huang et al., 2021; Biswal et al., 2022). The N-terminal intrinsically disordered tail is believed responsible for recruiting N protein into stress granules (Chang et al., 2014).

In the context of SARS-CoV-2 infection, Enoxaparin (EP) (a negatively charged, low molecular weight heparin) acted as an anticoagulant medication. The 2D CON NMR experiments in the presence of EP showed considerable chemical shift perturbation within the arginine-rich region of IDR1 ^37^SKQRRPQ^43^ and influenced the resonances in the IDR2 segment mainly composed of leucine residues ^217^AALALLLL^224^, by promoting this region to sample a helical conformation. Such changes do not describe this scenario as a direct interaction with a negative “ligand” but lead to the conclusion that interaction with EP disrupts intramolecular interactions, as also observed upon RNA binding (Schiavina et al., 2022).

Replication-transcription complex (RTCs) is one of the vital machinery that enables the virus to recruit proteins. In betacoronaviruses, N is the major cofactor of this complex that interacts with the amino-terminal ubiquitin-like domain of Nsp3 (Ubl1). The study of Bessa et al. revealed a bipartite interaction between Ubl1 and two motifs in the IDR2 linker of N a hydrophobic helix (^219^LALLLLDRLNQL^230^) and a disordered polar strand (^243^GQTVTKKSAAEAS^255^). These interactions induce the folding of portions ofIDR2 around Ubl1, that in turn brings conformational changes in N protein and regulates its RNA binding (Bessa et al., 2022). Thus, the intrinsically disorder regions are hypothesized to be important players in complex mechanisms that underlie the regulation of ribonucleoprotein complex formation.

Serine and arginine residues are highly disorder-promoting residues (Campen et al., 2008), and are the most abundant in the flexible intrinsic disordered linker region of all types of coronaviruses (Barik, 2020). Coronavirus N protein phosphorylation is a pivotal process for the viral life cycle that predominantly occurs within distinct regions of intrinsically disordered proteins. Primarily, N protein phosphorylation is processed within the SR rich intrinsically disordered region, both in host cells and virions (Chen et al., 2005; White et al., 2007). However, the mechanisms and potential function of multiple SR rich motifs are still inadequately comprehended (Yaron et al., 2022; Bouhaddou et al., 2020; Davidson et al., 2020). N protein interacts with many viral proteins, nucleic acid, and the host protein factors through specific and nonspecific mechanisms of interaction. Such interaction may be facilitated, at least in part, by the phosphorylation process occurring mainly in the rich SR amino acid region of the IDRs.

It has been reported that phosphorylated N-protein strongly affects the RNA binding specificity (Wu et al., 2021) and nucleocytoplasmic shuttling of the N proteins (Bai et al., 2021). Hyper-phosphorylation of N protein also plays a role in promoting the interaction between replication-transcription complex and viral replicase subunit Nsp3 (Keane and Giedroc, 2013). Notably, phosphorylation offers a mechanism for the N protein to recruit host factors like RNA helicases that promote viral RNA template switching and subgenomic mRNA transcription (Wu et al., 2014). The N protein is presumably engaged to host mRNA and G3BP1, thus playing a role in suppressing the host innate immune response. Additionally, even though the underlying mechanisms are not clearly known, the phosphorylation of the central disordered region promotes the protein transcriptional function (Carlson et al., 2020). Indeed, the indispensable role of intrinsically disordered regions in the phosphorylation processes is worth mentioning and more should be explored on their regulatory roles and the phosphorylation itself in modulating and expanding the repertoire of biological activities of N protein in SARS-CoV-2.

Therefore, taken together all the above highlighted hallmarks make IDRs of the N protein a pivotal avenue for SARS-CoV-2 therapeutic advancement.

### 3.2 Non-structural protein 1

The biological function and mechanism of activity of regulation of Nsp1 seems dependent on the interplay between the N-terminal folded domain and the unstructured C-terminal domain, which comprises IDRs that bind to the host ribosome.

The role of the N-terminal domain in translation inhibition of Nsp1 and the binding of SARS-CoV-2 Nsp1 to the stem-loop structure of RNA 5′-untranslated region (SL1) without the ribosome are two important topics. These SL structures are components of RNA 5′-untranslated region (5′UTR) that plays a role in regulation of gene expression, translation initiation, RNA folding, and stability. The SL1 region of the SARS-CoV-2 5′UTR is a highly conserved sequence necessary to evade Nsp1-mediated translational suppression (Vora et al., 2022; Lan et al., 2022).

**FIGURE 6:**
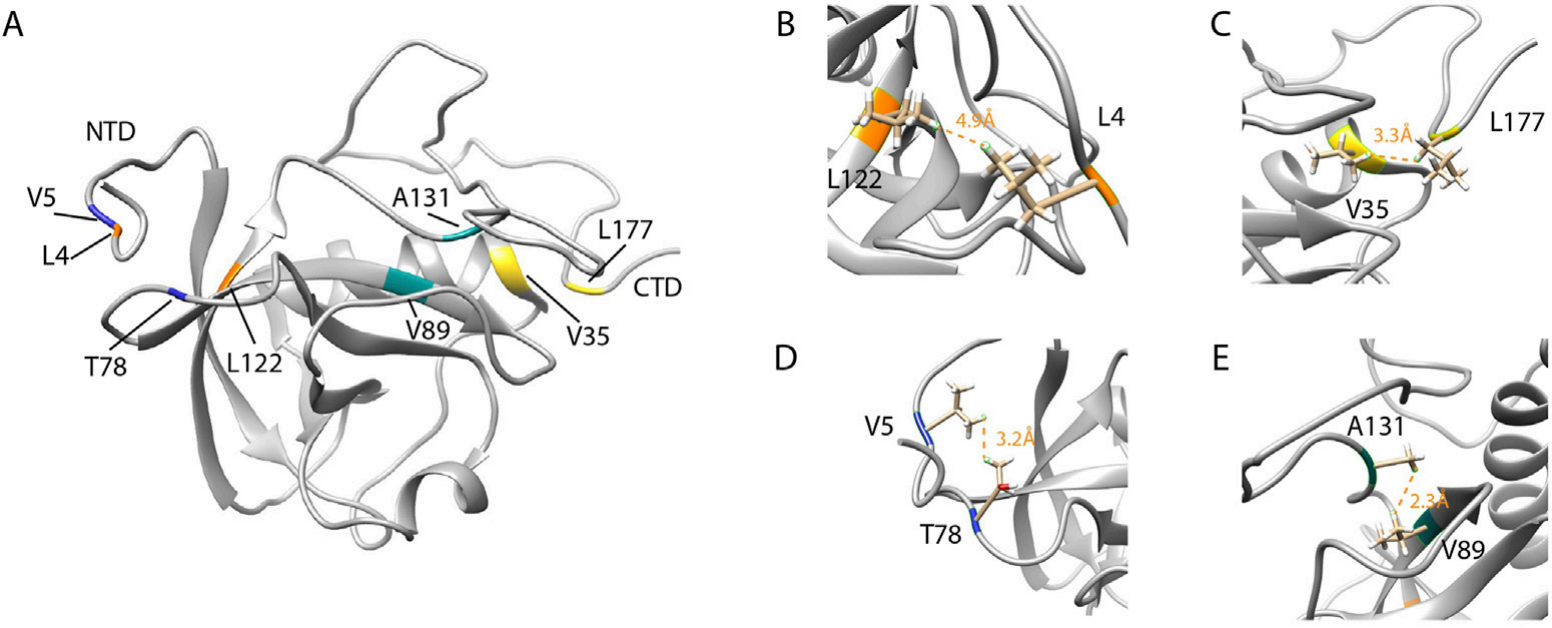
The interaction of the flexible C-terminal tail with the NTD of SARS-CoV-2 Nsp1. **(A)** SARS-CoV-2 Nsp1 mapping of residues that undergo NMR long range NOE contacts. **(B,D)** reports the residues L122 and L4 (orange) and V5 and T78 (blue) for which NMR long-range NOEs (orange dashed lines) between the short N-terminal tail and the NTD were observed and equals to 4.9 Å and 3.2 Å respectively. **(C and E)** show the residues V35 and L177 (yellow) and A131 and V89 (cyan) for which long-range NOEs between the NTD and the long C-terminal tail were detected and equals to 3.3 Å and 2.3 Å respectively. (PDB ID:8AOU). Adapted from Wang et al. (2023).

From solution NMR studies of SARS-CoV-2 Nsp1 (Agback et al., 2021), Agback et al. proposed intramolecular interactions between the disordered and folded Nsp1 domains. In fact, the Nsp1 H81P mutant shows chemical shifts perturbations within those parts of the protein that link structured/unstructured regions (amino acids 10-17 and 120-127). Similarly, other groups of researchers also suggested possible interactions between the disordered and folded Nsp1 domains (Figures 6A–E) (Wang Y. et al., 2021). These findings suggest that these domains are not fully independent units. Indeed, the folded NTD and the C-terminal intrinsically disordered region interact and their interaction is essential for the regulatory mechanism governing the activity of SARS-CoV-2 Nsp1. Molecular dynamics and simulation studies reported for SARS-CoV-2 the possible binding between SL1 and both the N-terminal globular and the C-terminal intrinsically disordered region of Nsp1 (Vankadari et al., 2020; Sakuraba et al., 2022): several interactions were observed between Nsp1 (residues including T12, Y118, R124, K125, N128, K129, L141, and D147) and SL1. In order to validate these findings, the residues K47, K58, R124/K125 were replaced with Alanine. Nsp1-wt, K47A, and K58A mutants were co-immunoprecipitated with the luciferase RNAs containing the 5′UTR. Interestingly, co-immunoprecipitation of R124A/K125A with the luciferase RNAs containing the 5′UTR was not detected, suggesting the crucial role of Nsp1 residues R124, K125 for viral RNA recognition (Tanaka et al., 2012; Terada et al., 2017; Mendez et al., 2021). From previous observations, it was assumed that the C-terminal domain of SARS-CoV-2 Nsp1 is necessary and sufficient to shut off host gene expression (Schubert et al., 2020). Later on, other studies based on *in vitro* and *in cell* experiments, further underlined that the coordinated mechanism of action including the N-terminal and central domains of Nsp1 are essential for host translation inhibition (Mendez et al., 2021; Wang et al., 2023). This could further strength the hypothesis on the molecular interplay among the folded domains and disordered regions.

Wang and coworkers solved the solution NMR structure of SARS-CoV-2 full-length Nsp1 (PDB ID: 8AOU) (Wang et al., 2023). They demonstrated, *via* 3D NOESY experiments, the interactions between the tails and the globular domain. As a result, it was pinpointed that the interplay between the folded NTD and the disordered C-terminal tail is required for the Nsp1 mechanism at the base of its regulation activity. For example, long-range NOEs were observed between the side chains of L4 and L122 and between V5 and T78. The unambiguous NOEs which were reproduced in all structures of the NMR ensemble indicate that residue L177 of the CTD contacts V35 of the core domain while in the side chains of NTD (Figure 6C), V89 contacts A131 of the linker region (Figure 6E), W161 of the flexible C-terminal tail contacts residues R43 and K125 of the N-terminal domain. This latter residue belongs to the conserved amino acid motif (LRKxGKG) that was found to be important for the RNA-binding activity of Nsp1. Moreover, the residues in the CTD that form helices interacting with the 40S ribosomal subunit in the Cryo-EM structure of Nsp1 (Schubert et al., 2020) are mostly disordered in free Nsp1. Thus, in the free form, C-terminal residues tend to interact with NTD, as an interaction was observed at W161 and L177 on the surface of the Nsp1 NTD (Wang et al., 2023). Another concept that seems controversial is the binding of SARS-CoV-2 Nsp1 to SL1 without the ribosome. Some studies suggest a 40S ribosome-mediated association between Nsp1 and the 5′UTR of viral mRNA (Mendez et al., 2021; Tidu et al., 2021); others also observed a direct interaction between SARS-CoV-2 5′UTR RNA and the Nsp1-NTD (Vora et al., 2022). However, Wang et al. clearly demonstrated that free Nsp1 has no binding affinity for both the full-length and stem-loop constructs of RNAs derived from the 5′UTR region of the SARS-CoV-2 genome. The authors further propose that the interaction of the C-terminal tail with the positively charged surface of the Nsp1 NTD may prevent non-specific RNA binding due to functional interplay between the two domains.

Wang et al. also uncovered the critical role of NTD in 40S-mediated viral mRNA recognition and promotion of viral gene expression. Nsp1 NTD, the linker, and the C-terminal tail are all involved in recruiting mRNA, further implicating that the intramolecular interaction and communication between the domains is required for the biological activity of Nsp1. Moreover, to validate the role of the linker, Shi et al. made Nsp1 constructs with different lengths of the linker between the Nsp1-N terminal domain and the Nsp1-C terminal domain. Accordingly, Nsp1 constructs with a linker domain extended beyond 20 residues were incapable of supporting viral translation, underscoring the significance of this spacer in the regulation of Nsp1 activity (Vora et al., 2022). Furthermore, upon binding of viral 5′ UTR to the Nsp1-NTD, the linker undergoes conformational changes that, in turn, significantly decrease the interaction of the CTD domain with the mRNA entry site of the 40S ribosome (Mendez et al., 2021; Tidu et al., 2021).

## 4 The role or association of IDP sites with variants of SARS-CoV-2 and infection

As discussed in the previous sections, the interaction of N-protein with the genomic RNA and other membrane proteins is a vital process for the assembly and release of new virions. To get insights into the evolution of drug resistance mutations, it is imperative to comprehend the mechanisms by which mutations are rectified by the virus. In this part, we summarize the contribution IDRs and IDPs across the SARS-CoV-2 genome in bearing mutations, modulating the protein functions and association with variants of SARS-Cov-2.

Genetic variations across the SARS-CoV-2 genome are associated with the frequency of variants, the transmissibility of the virus, and the anti-viral immune response of the host. Mutations in different proteins govern viral replication efficiency, transmissibility, and virulence properties. In the same scenario, mutations affect the properties of proteins and IDR patterns; they may increase as well the size of the IDRs, all of which have an essential effect on folding and SARS-CoV-2 pathogenesis. As IDRs mediate coronavirus transmission (Goh et al., 2020; Tomaszewski et al., 2020; Podder et al., 2021), offering also sequence and structural flexibility, they are believed critical for immune evasion and antibody escape (Quaglia et al., 2022; Li et al., 2024) through diversification and adaptation within its new host.

Several lines of experimental and computational studies were accomplished soon after the emergence of the pandemic, clearly demonstrating that expansion and frameshift of mutations, including the dominant variants of N and S proteins, mainly emerge across the intrinsic disorder regions in the SARS-CoV-2 proteome. This points out the vital role of IDPs in the emergence of new variants and higher transmissibility than previous SARS‐coronaviruses (Quaglia et al., 2022; Feng et al., 2024; Fang et al., 2021).

The infection of SARS-CoV-2 in humans requires S proteins, which bind to the human ACE2 proteins. Particularly, several disordered regions in the receptor binding domains (RBDs) of Sprotein facilitate the interaction (Yesudhas et al., 2021). Multiple studies observed numerous pairwise interactions within unstructured regions while examining the atomic interaction between SARS-CoV-2 and ACE2 (Bhattacharjee et al., 2021; Celik et al., 2021; Michelitsch and Weissman, 2000; Campbell et al., 2021).

The mode of transmission and rigidity of the coronavirus outer shell appeared to be influenced by the unstructured region of Membrane and Envelope proteins (Goh et al., 2020), confirming the strong association between variants of SARS-CoV-2 and its intrinsically disordered regions (Quaglia et al., 2022; Gomari et al., 2023; Sinha and Roy, 2023; Ye et al., 2021).

From the analysis of 219.909 SARS-CoV-2 N sequences, most mutations localized in the intrinsically disordered regions, particularly in the SR-rich linker region (Zachrdla et al., 2022), that were as such believed to be the culprits in increasing the spread of the SARS-CoV-2 virus (Johnson et al., 2022; Rahman et al., 2021).

At the early stages of the SARS-CoV-2 outbreak, the increased spread of variants, including B.1.617.2 (Delta), which contains the R203M mutation has been noticed. Later on, this mutation (R203M) in N and D614G in S were documented along with the other six mutations (P323L in Nsp12, D614G in S, the T492I in Nsp4, R203M in N, T60A in Orf9b, and P1228L in Nsp3) that had greater than 50% frequency in the global SARS-CoV-2 mutations (Abbasian et al., 2023).

In a general context, the diversity of structural ensembles for proteins and protein-protein complexes can be explained by the major concepts of frustration and fuzziness. As illustrated in Figure 5, researchers used the terms frustration to describe conformational variations in proteins and fuzziness to describe how conformational heterogeneity occurs in protein complexes involving IDRs (Freiberger et al., 2021; Gianni et al., 2021). In this regard, Haque et al. analyzed 228 mutations from each domain of the N protein of the SARS-CoV-2. Accordingly, 11 mutations are predicted to be deleterious and destabilizing. Among these mutations, R32C, R191C, and R203 M mutations are localized in the disordered regions and show a significant change in frustration state (from neutral frustrated to major minimally frustrated state), which could raise the sampling of conformation space (Haque et al., 2023). It is worth noting that the R203M mutation improves RNA packaging mechanism and production of higher titers of infectious virions (Haque et al., 2023; Syed et al., 2021).

Phosphorylation of the SARS-CoV-2 Nucleoprotein recruits human cytosolic 14-3-3 proteins, essential for virus replication. 14-3-3 isoforms have the highest expressed rate in a number of tissues, including the brain, lungs, and gastrointestinal system. A recent study predicts the effect of N mutations on the interaction of N protein with human cytosolic 14-3-3 proteins. The S202R mutation demonstrates the profound effect on the efficiency of N protein to package RNA. R203K/G204R mutations, that occurred in the IDRs, appear to enhance the interaction with the 14-3-3 binding motif due to a predicted Arg204:Asp225 salt bridge (Tugaeva et al., 2023). Moreover, it was observed that the arginine at site 203 decreased in prevalence by 8.33% (from 81.9% to 73.5%) as the incidence of a lysine variant increased by 8.2% (from 18.12% to 26.32%). The glycine residue of the directly adjacent 204 site was exchanged by an arginine, increasing of 8.2% the incidence of the mutation (from 18.1% to 26.3%) (Tomaszewski et al., 2020). These results underscore the importance of these specific mutations in contributing to the function of the disordered SR rich region of the protein. Notably, SR rich region in the IDR2 of the N protein that includes the R203K mutation was the hot spot harboring 7 of the top 10 most frequent mutations (Zhao et al., 2021). Mutant proteins bearing mutations in these regions resulted in noticeably weaker phase separation with respect to the wild-type N protein. As confirmed by deep sequence data analysis of SARS-CoV-2 from public databases and clinical samples, the R203K/G204R variant was also associated with novel transcription-regulating sequence-core sequence-dimerization domain RNA, that is supposed to have an increased effect on the expression of sub-genomic RNA (Leary et al., 2021).

Another important aspect of mutations is their role in modulating the viral-host protein-protein interaction. Interestingly, Del Veliz et al. suggested the presence of compensatory mutations in N disordered regions containing binding motifs for 14-3-3 proteins. From analysis of the variability of about 90, 000 sequences of the SARS-CoV-2 N protein, it has been observed that most residues were highly conserved within the 14-3-3 binding site. Moreover, the compensatory mutations, including half of the variants of concern, were found to maintain the interaction energy of N-14-3-3γ which is suggested to be beneficial for the virus (Del et al., 2021).

Protein-protein interactions are initiated by molecular recognition features (MoRFs) (Mohan et al., 2006; Kotta-Loizou et al., 2013), the abundance of which is highly associated with the amount of intrinsic disorder in different forms of life (Yan et al., 2016). MoRFs are small disordered regions situated within intrinsic disorder proteins or regions that undergo a disorder-to-order structure arrangement during interaction with their partners (Gypas et al., 2013; Mishra et al., 2018).

Short linear motifs often located in intrinsically disordered regions offer a mechanism to hijack a large number of host processes (Van Roey et al., 2013; Van Roey et al., 2012). In SARS-CoV-2, nearly all proteins are predicted to have a significant percentage of MoRFs (Giri et al., 2021) suggesting the role of IDRs in protein-protein interaction networks. It has been observed that the replacement of hydrophobic to polar and charged amino acids in S glycoproteins of SARS‐CoV-2 creates an intrinsically disordered region that facilitates proteolysis and fusion with the host membrane (Podder et al., 2021). The antigenicity of the SARS-CoV-2 is due to the structural flexibility of its surface proteins. In S glycoproteins, mutation‐driven accumulation of intrinsically disordered residues has been noticed and hypothesized to enhance viral transmissibility. This overlap between IDRs and major antigenic sites in the receptor-binding domain and N-terminal domain of S glycoprotein (S1 subunit) (Quaglia et al., 2022) is further implicated in the virus to provide genetic and antigenic drift.

While the mutations that originated in the intrinsically disordered regions improved the viral fitness and affected the interaction with the human proteins, it is possible that they influence protein folding and glycosylation leading to structural modifications. Studies by Fang et al. reported nonsynonymous single nucleotide variants (SNVs) on more than 60% of each S, ORF1ab, and ORF3a, with significantly higher SNV occurrence ratios in IDRs of S and ORF1ab. The authors observed mutations in the IDRs of S located near N‐linked glycan sites, postulating potential impacts on glycosylation and protein folding, as well as on elevated levels of hydrophobicity (in S, ORF1ab, but not in ORF3a) (Fang et al., 2021).

Single mutations can affect the intrinsic disorder predisposition of viral proteins. In their per-residue disorder analysis, Hassan et al. used predisposition scores from 0 to one for the per-residual conditions, where 0 and one indicate residues entirely arranged and completely disordered, respectively. Accordingly, with different degrees of disorder predisposition, they observed that single mutations had a pronounced effect on the intrinsic disorder predisposition of ORF10 at the N- and C- terminal of its regions (Hassan et al., 2022). Mutations that emerge on the intrinsic disordered regions, could also lead to the development of drug and vaccine resistance. For example, as compared to wild type, receptor-binding domain mutants of Omicron, Delta AY.1, AY.2, and AY.3 were determined to be more disordered (Ranjan et al., 2022). This increase in disorder could perturb the interactions with antibodies, thus favoring the virus for antibody escape and, as a consequence leading to more pathogenicity.

It is also possible that mutations at the IDRs may lead to structure destabilization. For example, both SARS-CoV-2 and SARS-CoV-1 deletion and substitution mutations of Nsp1 in the C-terminal motif IDR significantly destabilize the structure of Nsp1 (Rezaei et al., 2022). In fact, point mutations at R99 and residues Arg124-Lys125 near the predicted unstructured regions of Nsp1 also lead to the loss of its ability to recognize viral RNA (Mendez et al., 2021; Lokugamage et al., 2012). Also interestingly, K129E and G130R mutations at the beginning of the intrinsically disordered C-terminal domain strongly reduced the ability of SARS-CoV-2 Nsp1 to inhibit cellular translation (Frolov et al., 2023).

## 5 SARS-CoV-2 IDRs and signaling pathways of the host cell

The unusually large genome of coronaviruses provides high structural complexity and intricate molecular interaction to efficiently replicate their genome. Once inside the infected cells, a spatiotemporal-oriented coordination between a cohort of enzymes, supporting proteins, and the host multiple factors, is required to produce viral mRNAs and new genomes. All stages of the life cycles of SARS-CoV-2 rely on host factors (Kim et al., 2020; Zhou et al., 2020), and hundreds of human proteins have been found to interact with viral proteins (Bouhaddou et al., 2020; Davidson et al., 2020; Gordon et al., 2020; Li et al., 2021).

In their large-scale proteomic analysis, Gordon et al. identified SARS-CoV-2 proteins interacting with human proteins involved in innate immune pathways, host translation machinery, cullin ubiquitin ligase, and bromodomain proteins (Gordon et al., 2020). Li and coworkers also identified 286 potential host targets using quantitative proteomic analysis approaches on SARS-CoV-2 intra-viral protein-protein interactions and virus-host interaction networks in human cells (Li et al., 2021). These findings further outline how the host cell infrastructure and metabolism ultimately determine the viral progeny.

In this section, we emphasize the importance of IDRs in SARS-CoV-2 by scrutinizing biochemical, structural, and virological experimental studies of selected viral proteins (Table 1), whose function includes IDRs/IDPs involvement in the host signaling machinery. However, the complete molecular mechanism of interaction of the virus protein with host proteins is not mentioned here because it is outside the scope of this review.

**TABLE 1:** Some selected examples of the potential involvement of intrinsically disordered regions of SARS-CoV-2 proteins in host signaling machinery.

Viral components/IDR	Unstructured Region	IDR length/location	MoRF motif sequence^a^	Experimental assay	Role in host machinery	Change/response/effect on the host	References
**N**	Mostly in the N terminus	Residues 1–48, 175–248, 365–419	^1^MSDNGPQNQRNAPRITFGGPSDSTGSNQNGER^20^, ^82^DQIGYYRRATRRIRGGD^98^, ^102^KDLSPRWYFYYLGTGPE^118^, ^403^FSKQLQQ^409^	Analytical size-exclusion chromatography (SEC)	Interacts with seven human 14-3-3 isoforms^c^	Could hijack cellular pathways by 14-3-3 sequestration	Del et al. (2021), Tugaeva et al. (2021)
				*In vitro* host-SARS-CoV-2 interactome, Y2H and TMT-AP–MS	Interacts LARP1 and RRP9	Downregulation of LARP1 phosphorylation Inhibition of protein synthesis	Gordon et al. (2020), Zhou et al. (2023)
**ORF6**	Mostly the C-terminal^a^	Residues 45–60	^14^ILLIIMRT^21^, ^26^IWNLDYIINLIIKNLSKSLTENKYSQLDEEQPMEID^61^	*In vitro* host-SARS-CoV-2 interactome	Interacts with NUP98/RAE complex	Prevents host mRNA export through the nuclear pore	Gordon et al. (2020)
**ORF3a**	N-terminus, and C-terminus^a^	Residues 1–19, 261–268	^1^MDLFMR^6^, ^261^EPIYDEPT^268^	*In vitro* host-SARS-CoV-2 interactome, Y2H and TMT-AP–MS	Binds TRIM59 and other HOPS complex proteins	Endoplasmic reticulum stress	Gordon et al. (2020), Li et al. (2021); Zhou et al. (2023)
**Nsp1**	C-terminal	Residues 148–180	^64^LEQPYVFIKRSDA^75^, ^93^EGIQ^97^, ^99^RSGETLGVLVPHVGEIPVAYRKVLLRKNGNKGAGGHSYGADLKSFDLGDELGTDPYEDFQENWNTKHSSGVTRELMRELNG^179^	Cryo–electron Microscopy, *In vitro* binding assay	Interacts with 40s ribosome	Mediates translation shutoff	Thoms et al. (2020), Frolov et al. (2023)
**Nsp8**	Mostly at the middle (residues 40–84^a^)	Residues 40–84	^181^AWPLI^185^	*In vitro* host-SARS-CoV-2 interactome	Interacts with SRPs components (SRP19, SRP54, SRP72)	Hijacks the Sec61-mediated protein translocation pathway for entry into the endoplasmic reticulum	Gordon et al. (2020)

^a^ The disorder prediction results, and.

^b^ the predicted MoRF, motif sequences results are from Giri et al. (2021). It should be noted that Giri et al. used several MoRFs, predictors that may show some variation in the results, and the MoRF, regions presented here in Table 1 are drawn from the MoRFchibi_web predictor. The C-terminal sequence of SARS-CoV-2 ORF6, contains a trafficking motifs 46ENKYSQLDEEQPMEID61, and putative NUP98–RAE, binding sequence^56^ QPMEID61 Gordon et al. (2020).

^c^ A study shows that greater than 90% of 14-3-3 protein partners contain disordered regions, suggesting that intrinsically disordered regions are favored by 14-3-3 proteins Bustos and Iglesias (2006).

Abbreviations: SRP, signal recognition particle; HOPS, homotypic fusion and protein sorting complex; LARP1, La-related protein 1; Y2H, yeast two-hybrid; TMT-AP–MS, tandem mass tag affinity purification followed by mass spectrometry.

As was said earlier, intrinsically disordered protein regions evolve rapidly, promoting diversity and enabling interaction with multiple proteins among species (Ortiz et al., 2013; Chang et al., 2014) (Gitlin et al., 2014)*.* In SARS-CoV-2, ORF6 is one of the second-highest IDR-containing accessory proteins. ORF6 hijacks nucleocytoplasmic mRNA transport factors Nucleoporin 98 (NUP98) and RNA export factor 1 (RAE1) (Miorin et al., 2020; Miyamoto et al., 2022; Kato et al., 2021). Using its C-terminal residues directly binds to transcription factors, signal transducers, and activators of transcription (STAT1) and inhibits its nuclear localization, therefore changing subcellular localization and resulting in downregulation of the interferon (IFN) pathway (Miyamoto et al., 2022; Lei et al., 2020). Importantly, this protein contains a long MoRF region (residues 26–61) near the C-terminal region (Giri et al., 2021), that matches with the residues 46–61 comprising trafficking motifs and with putative NUP98–RAE1 binding sequence (Gordon et al., 2020).

### 5.1 Nucleocapsid and host cell interaction

Several lines of experimental and computational studies revealed that the intrinsic disorder regions of N protein contain putative binding sites for different host proteins (Jia et al., 2022; Huang et al., 2021; Biswal et al., 2022; Del et al., 2021; El-Maradny et al., 2024). As a result of a recent investigation, 77 coronavirus N‐interacting host proteins were examined, of which 33 are stress granule components, indicating a higher likelihood of IDRs to hijack SG proteins to the viral replication machinery (Moosa and Banerjee, 2020). It has been implicated that viral N‐mediated host subversion is associated with π‐π‐interactions between π‐rich disordered domains of coronavirus N and host proteins. This bioinformatics report is supported by experimental observations on the possible self‐association of π‐rich IDR‐2 *via* π‐π interactions (Cong et al., 2017). Likewise, IDR‐1 also promotes self‐interaction of SARS N (He et al., 2004). Furthermore, there is also a tight interaction of the π‐rich IDR‐2 of coronavirus N with the π‐rich disordered domain of heterogeneous nuclear ribonucleoprotein A1 (hnRNPA1) (Luo et al., 2005), all of which interactions contributes to suppress the host gene expression.

To fully understand the role of N-terminal IDR1, Huang et al., by means of GST pull-down and ITC assays, showed the key fragments for interaction between G3BP1/2 and N protein: the binding is mediated by N-IDR fragment (amino acids 11-24) and the NTF2 domain from the G3BP1 (G3BP1^NTF2^) (Huang et al., 2021). Another important finding regards the sequence termed ITFG motif, found in the N-IDR (residues 1–48) region of the N protein. The motif shares similarity with that of a peptide known to interact with G3BP1^NTF2^ and fully conserved in SARS-CoV: interestingly, all four residues contribute to the binding, with Isoleucine and Phenylalanine paramount. Furthermore, from truncation of the N protein, it was shown that both the N-IDR1 and N-terminal RNA recognition motif RRM (residues 49–174) are required for inhibiting G3BP1-mediated LLPS (Huang et al., 2021). From another standpoint, the C-terminal domain (spanning residues from 248 to 365)-mediated inhibition of SG formation is not affected by disruption of the G3BP binding motif. On the other hand, alanine substitution in the C-terminal domain (248–365), as, for example, K257A, K261A, and their combination, disrupt dsRNA binding and abrogate suppression of SG formation (Aloise et al., 2023).

### 5.2 Nsp1 and host cell interaction

Nsp1 interactions with human proteins are indispensable for the virus life cycle. The immune evasion and the control of cellular response relies on host mRNA degradation and translation shutoff. The major virulence factor, Nsp1, governs this process *via* direct ribosomal association (Schubert et al., 2020; Vora et al., 2022; Lokugamage et al., 2012; Yuan et al., 2020).

Nsp1 N-terminus domain of both SARS-CoV-1 and SARS-CoV-2 binds the first stem-loop in the viral 5′leader sequence that guarantees continuous translation of the viral mRNA (Tanaka et al., 2012; Vora et al., 2022). Multiple structural and biochemical studies on coronaviruses demonstrated the C-terminal region as a key domain of Nsp1 that interacts with different ribosome subunits (Schubert et al., 2020; Kami et al., 2009; Huang et al., 2011; Nakagawa et al., 2016). Structural analysis by Cryo-electron microscopy evidenced that Nsp1 interacts with the 40S ribosomal subunit *via* residues conserved among coronaviruses, namely, Lys164 and His165. Thus, it leads to mRNA entry tunnel obstruction by C-terminus attachment. In consequence, the antiviral defense factor including RIG-I (retinoic acid-inducible gene-I) is suppressed by Nsp1. In fact, K164A and H165A mutants do not show binding, explaining the role of the two residues in the interaction with the 40S ribosome in both SARS-CoVs (Thoms et al., 2020; Schubert et al., 2020). Moreover, in the case of molecular interactions between Nsp1 and the 40S subunit, residues Y154, F157, and W161 in the first C-terminal helix (residues 153–160) interact through their multiple hydrophobic side chains with uS5 and uS3 proteins (Schubert et al., 2020).

It is also observed that the conserved KH motif (i.e., K164 and H165 in the short loop) experiences stacking interactions with helix h18 of the 18S rRNA *of the 40S subunit*, thereby mediating the interaction in a conserved manner. The two C-terminal helices (i.e., the first helix (residues 153–160) and the second helix (residues 166–178) are connected by the KH motif. The other two conserved arginine R171 and R175 in the second helix make interactions with the phosphate backbone of h18 (Schubert et al., 2020). The uS3 subunit, which most likely corresponds to the Nsp1 unstructured linker, weakly interacts with h16. Furthermore, the C-terminal Nsp1 binds also 80S ribosomal complexes proving its role in the global translation inhibition (Schubert et al., 2020).

As highlighted in the previous sections, the efficient interplay between the NTD and C-terminal unstructured region is fundamental for Nsp1 interaction with host ribosomes (Wang et al., 2023).

## 6 IDR-targeted therapeutics and future perspectives

IDPs have gained great attention and opened new insights for understanding the molecular mechanism of diseases beyond the classic structure-function paradigm. Nowadays, the structural and functional characterization of IDPs and IDRs is becoming essential and thus unveiling the roles of protein disorder in protein-protein and protein-nucleic acid interactions is a promising field in understanding the biology of many diseases. Thus, understanding viral IDPs/IDRs and their molecular recognition features is essential for grasping their role in protein function and drug design, especially in light of the fact that SARS-CoV-2 variants arise within intrinsically disordered regions (Kulkarni et al., 2025; Gupta and Uversky, 2024).

One of the SARS-CoV-2 proteins bearing the highest content of intrinsically disordered regions is the multifunctional nucleoprotein N. Its major biological function depends on the interplay between the folded domains and the intrinsically disordered regions. Being the N protein a RNA packaging protein, the importance of its disordered regions in RNA binding and as major phosphorylation site, leads to consider the IDRs of the N protein potential areas for future studies to develop therapeutics. As such, though the SR-rich intrinsically disordered region is identified as a preferential binding site to human 14-3-3 protein (Del et al., 2021), little is known about the fundamental biology of this region.

Thus, the host-viral interacting IDPs/IDRs could be targeted for different therapeutic strategies.

Indeed, the survival of the virus inside the host cell is guaranteed by the dynamic molecular interactions of intrinsically disordered regions of Nsp1 and host ribosome subunits; this protein hijacks the host ribosome, through the intrinsically disordered C-terminal domain and thereby leads to host gene shutoff. The fully comprehension of the Nsp1 role in SARS-CoV-2 pathogenesis definitely needs the characterization of Nsp1 structural and dynamical properties along with its molecular interactions.

Gordon et al. reported a comprehensive SARS-CoV-2 PPI network, and the drug-human target network, suggesting that SARS-CoV-2 proteins, such as Nsp8 and ORF6, whose interaction sequence comprises the IDR, show potential druggable targets (Gordon et al., 2020).

Mutations can significantly affect the physicochemical properties of proteins. For instance, it has been shown that mutations in the IDRs of N protein can have nonlocal impact and modulate thermodynamic stability, secondary structure, particle formation, protein oligomeric state, and LLPS (Nguyen et al., 2024). From another perspective, experimental studies demonstrated the impact of the spike protein D614G mutation that induces IDR-like segments to form “order–disorder” transition (Zhang et al., 2021; Dokainish and Sugita, 2023). It has been further revealed that the order-disorder transition of IDRs in VOCs, which are associated with characteristic mutations such as D614G in the proximity of IDRs, controls the spike structure and dynamics (Sinha and Roy, 2023). This scenario opens an opportunity to target IDRs as remote operational switches for alternative therapeutic interventions. Moreover, as discussed above, IDRs show a helical propensity that possibly forms druggable conformational states. Interestingly enough, a recent study revealed that a small molecule to be evaluated in a clinical trial selectively interacts with an IDR oligomerization, further offering an insight that condensation and oligomerization could serve as key factors in the development of inhibitors for IDRs (Bielskutė et al., 2025).

Overall, although the presence of disordered regions could explain difficulties with crystallization of full-length N and Nsp1 proteins, deciphering the structural nuances of these proteins can provide important clues about potential therapeutic targets. Therefore, future studies should be oriented toward understanding the detailed mechanisms of intra- and intermolecular interactions of IDRs that may help to guide structure based therapeutics against SARS-CoV-2.
